# Therapeutic effects of tetrahedral framework nucleic acids and tFNAs‐miR22 on retinal ischemia/reperfusion injury

**DOI:** 10.1111/cpr.13695

**Published:** 2024-07-31

**Authors:** Xiaoxiao Xu, Yanyan Fu, Delun Luo, Lina Zhang, Xi Huang, Yingying Chen, Chunyan Lei, Jinnan Liu, Shiqi Li, Zhouyuan Yu, Yunfeng Lin, Meixia Zhang

**Affiliations:** ^1^ Department of Ophthalmology, and Research Laboratory of Macular Disease, West China Hospital Sichuan University Chengdu China; ^2^ Innovative Institute of Chinese Medicine and Pharmacy Chengdu University of Traditional Chinese Medicine Chengdu China; ^3^ National Engineering Research Center for Biomaterials, College of Biomedical Engineering Sichuan University Chengdu China; ^4^ State Key Laboratory of Oral Diseases, West China Hospital of Stomatology, Department of Maxillofacial Surgery, West China Stomatological Hospital Sichuan University Chengdu China

## Abstract

Retinal ischemia/reperfusion injury (RI/R) is a common pathological process in ophthalmic diseases, which can cause severe visual impairment. The mechanisms underlying RI/R damage and repair are still unclear. Scholars are actively exploring effective intervention strategies to restore impaired visual function. With the development of nucleic acid nanomaterials, tetrahedral framework nucleic acids (tFNAs) have shown promising therapeutic potential in various fields such as stem cells, biosensors, and tumour treatment due to their excellent biological properties. Besides, miRNA‐22‐3p (miR‐22), as an important regulatory factor in neural tissue, has been proven to have positive effects in various neurodegenerative diseases. By stably constructing a complex of tetrahedral framework nucleic acids miR22 (tFNAs‐miR22), we observed that tFNAs‐miR22 had a positive effect on the repair of RI/R injury in retinal neural tissue. Previous studies have shown that tFNAs can effectively deliver miR‐22 into damaged retinal neurons, subsequently exerting neuroprotective effects. Interestingly, we found that there was a certain synergistic effect between tFNAs and miR‐22. tFNAs‐miR22 can selectively activated the ERK1/2 signalling pathway to reduce neuronal apoptosis, accelerate cell proliferation, and restore synaptic functional activity. In this study, we established a simple yet effective small molecule drug for RI/R treatment which may become a promising neuroprotectant for treating this type of vision impairment disease in the future.

## Graphical abstract

tFNAs‐miR22 could activates ERK1/2 pathway, modulating RGCs associated proteins (RPBMS and SYN) and neuroprotective genes (FTL, NET1, NUS1), while balancing anti‐apoptotic (Bcl‐2) and pro‐apoptotic (Caspase‐3, BAX) proteins, demonstrating therapeutic potential in retinal injury repair via targeted nucleic acid delivery, offering new strategies for Retinal ischemia/reperfusion injury treatments.

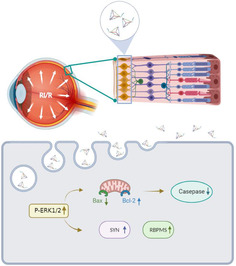

## 1 INTRODUCTION

RI/R injury is one of the common causes of visual impairment and blindness.^1^ The retina is a nerve tissue supplied by the terminal vascular system. Any pathological damage and tissue ischemia caused by vascular obstruction can lead to tissue necrosis, often resulting in irreversible visual impairment within a short period of time.^2^ Several ocular diseases involve this pathological process, including central retinal artery occlusion (CRAO),^3^ acute angle‐closure glaucoma (ACG),^4^ anterior ischemic optic neuropathy (AION),^5^ diabetic retinopathy (DR),^6^ and so on. Based on the secondary injury mechanism of RI/R, these diseases share a common feature: after reperfusion of retinal blood flow, the original damage not only fails to alleviate but continues to worsen, leading to further decline in visual function.^7^ Previous studies^8^ have shown that the main reason for visual dysfunction is the progressive loss of retinal ganglion cells (RGCs) following RI/R injury. Therefore, current interventions mainly focus on combating the death of RGCs.^9^ In vitro experiments have demonstrated that these interventions can partially prevent or delay the death of RGCs. However, due to the acute and severe injuries caused by RI/R, these interventions do not perform well in in vivo experiments.^10^

With the progress in the biomedical materials, DNA origami and DNA nanostructures has rapidly developed and been widely applied. After four stages of renewal iterations,^11^ the currently typical structure is tetrahedral frame nucleic acids (tFNAs), which are composed of four equidistant single‐stranded DNAs. Compared to traditional drug delivery systems, tFNAs have the following advantages^12^: ① simple synthesis method; ② high mechanical strength; ③ better stability; ④ editability; ⑤ good biocompatibility; ⑥ can regulate biological behaviour. Due to their excellent mechanical and biological properties, tFNAs have been successfully applied in various domains including stem cells, biosensors, and tumour therapy, establishing them as one of the most promising nucleic acid nanomaterials within the realm of life sciences.^13^

tFNAs have the capability to deliver a diverse range of small molecule drugs, including peptides, oligonucleotides, and miRNAs.^14^ Among these molecules, miRNAs are a group of endogenous non‐coding RNA sequences that exhibit widespread expression in the nervous system.^15^ Studies^16^ has indicated that miRNAs play crucial roles as regulatory factors by modulating gene expression at the transcriptional level and influencing cellular responses to external stimuli. Moreover, they may also contribute to the pathogenesis of RI/R.^17^ The protective effect of miRNA‐22‐3p (miR22) against neurodegenerative diseases such as Alzheimer's disease and Huntington ‘s disease has been experimentally validated.^18^ Furthermore, in vitro studies have demonstrated that the upregulation of miR22 in brain tissues of rats with Alzheimer's disease leads to a downregulation of expressions of brain‐derived neurotrophic factor (BDNF) and apoptosis‐related proteins.^19^ Transmission electron microscopy (TEM) results have revealed a significant improvement in both morphology and activity of synaptic structures through overexpression of miR22.^20^ Additionally, bioinformatic analysis has indicated that the DNA sequence corresponding to miR22 is one of the target genes for glaucoma therapy,^20^ highlighting its potential therapeutic value for RI/R diseases.

However, the unstable structure of single‐stranded miR22 has restricts their extensive application in research. In contrast, tFNAs system carrying miR22 (tFNAs‐miR22) can ensure the stability and activity of miR22 and accurately express in retinal neurons.^19^ Professor Lin's team pointed out in their research on facial nerve injury that tFNAs‐miR22 can selectively activate the ERK1/2 signalling pathway, thereby inhibiting neuronal apoptosis.^21^ The ERK1/2 signalling pathway mainly regulates biological processes such as cell proliferation, differentiation, survival, and the cell cycle.^22^ In addition, the ERK1/2 pathway can influence the expression of retinal‐related and nerve‐related factors and proteins and closely associated with various ophthalmic diseases,^23^ and studies have found that it plays an important regulatory role in anti‐inflammatory and anti‐apoptosis processes.^24^ However, the underlying mechanism through which tFNAs‐miR22 exerts a protective role in RI/R injured RGCs remains elusive.

**FIGURE 1:**
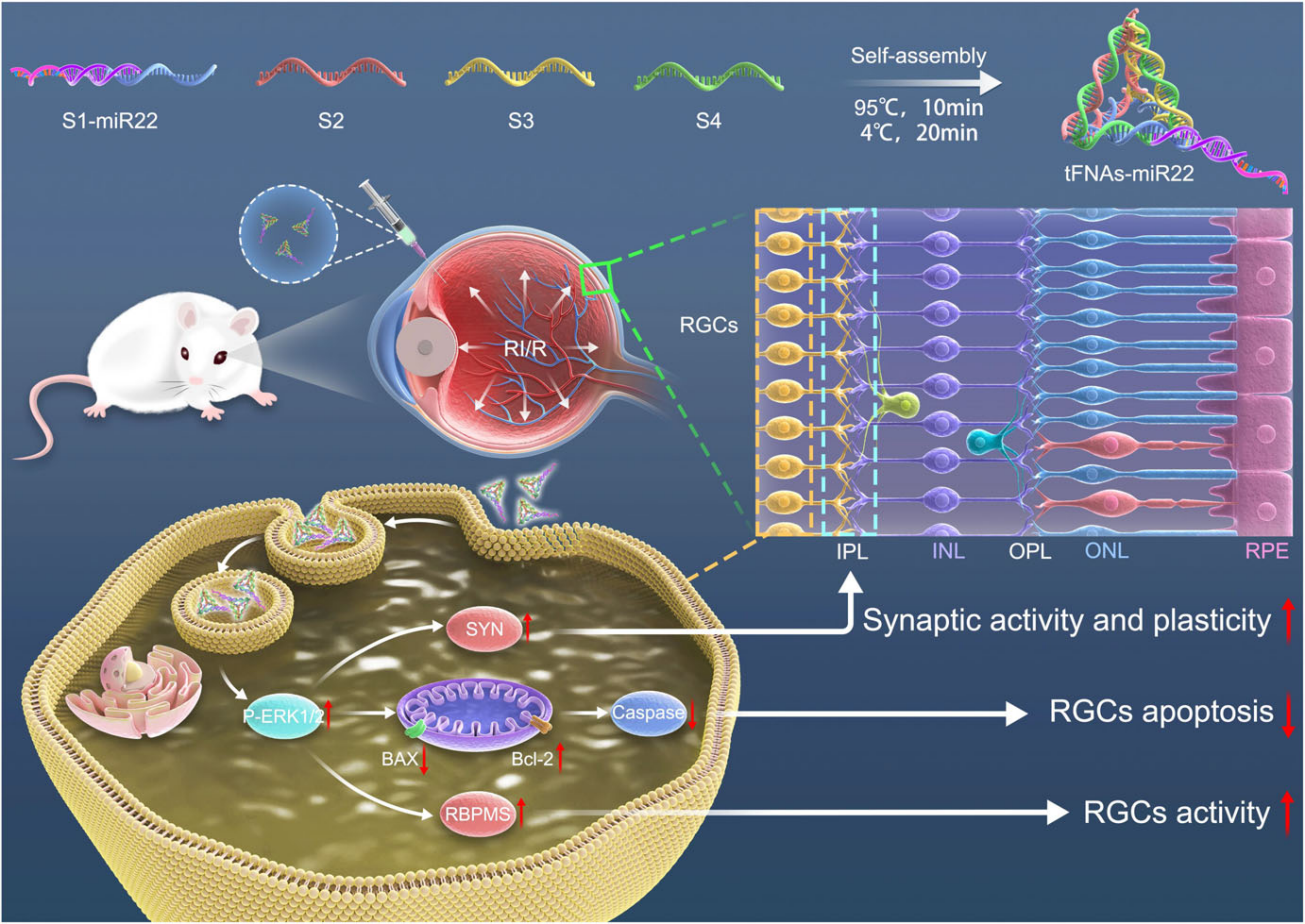
Schematic diagram of the mechanism by which tFNAs‐miR22 acts on I/R injured RGCs. The figure shows the intracellular protective mechanism of tFNAs‐miR22 after endocytosis into injured RGCs. Firstly, tFNAs‐miR22 was recognized and bound by the fusion protein on the cell membrane, and then entered the cell interior by endocytosis. Intracellular tFNAs‐miR22 activates the ERK pathway by phosphorylating ERK1/2, and also affect the expression of SYN and RBPMs, and enhance the viability of RGCs and the activity of synapses. In addition, tFNAs‐miR22 can regulates the balance of Bax (pro‐apoptotic protein) and Bcl‐2 (anti‐apoptotic protein) thereby preventing cell apoptosis. In conclusion, tFNAs‐miR22 plays a protective role inside RGCs thereby reducing the degree of RGCs damage and promoting their recovery and regeneration.

Based on the above experimental evidence, we anticipate that tFNAs possess the capability to deliver miR22 into RI/R injured RGCs stably and effectively, thereby exerting subsequent neuroprotective effects. Additionally, we have also investigated the underlying mechanistic aspects of tFNAs‐miR22, encompassing their potential to activate the ERK1/2 signalling pathway. This activation can inhibit apoptosis by regulating apoptotic protein expression, and promote proliferation by affecting the cell cycle, influence synaptic activity and plasticity for restoring RGC functionality. Moreover, it is noteworthy that the ERK1/2 signalling pathway can regulate transcription factor activity to impact gene expression related to both apoptosis and neuroprotection (Figure 1). This study will combine an RI/R injured rats model with an oxygen–glucose deprivation/recovery (OGD/R) cell model of RGCs to investigate the expression profile of tFNAs‐miR22 after injury as well as its therapeutic intervention efficacy. Our research endeavours to partially elucidate the protective mechanisms of tFNAs‐miR22 in RI/R injury and provide novel insights for retinal injury treatment.

## 2 MATERIALS AND METHODS

### 2.1 Preparation and characterization of tFNAs and tFNA‐miR22

#### 2.1.1 Preparation

First, we designed single‐stranded DNAs (Table 1) which were synthesized and characterized by Genescript (Nanjing, China). The four ssDNA strands were mixed at equal concentrations and added to TM buffer. After thorough mixing and centrifugation, the solution was heated to 70°C for 10 min and then rapidly cooled to 4°C for 20 min, resulting in the formation of tFNAs and tFNAs‐miR22. These samples obtained after synthesis were retained as pre‐purification sample and introduced into AKTA Pure150 protein chromatography system. Subsequently, tFNAs and tFNAs‐miR22 were purified using a DNA PacTMPA100 anion exchange column, employing different mobile phases (mobile phaseA: 25 mM Tris–HCl, mobile phaseB: 25 Mm Tris–HCl + 375 mM NaClO_4_). The purity of the main peak was evaluated using high‐performance liquid chromatography (HPLC).

**TABLE 1:** The sequences of the four single‐stranded DNAs (ssDNAs).

ssDNA	Base sequence (5′→3′)
S1‐miR22‐3p	**AGCUGCCAGUUGAAGAACUGU**‐TTTTT‐ACTACTATGGCGGGTGATAAAACGTGTAGCAAGCTGTAATCGACGGGAAGAGCATGCCCATCC
S1	ACTACTATGGCGGGTGATAAAACGTGTAGCAAGCTGTAATCGACGGGAAGAGCATGCCCATCC
S2	ACATGCGAGGGTCCAATACCGACGATTACAGCTTGCTACACGATTCAGACTTAGGAATGTTCG
S3	ACTACTATGGCGGGTGATAAAACGTGTAGCAAGCTGTAATCGACGGGAAGAGCATGCCCATCC
S4	ACGGTATTGGACCCTCGCATGACTCAACTGCCTGGTGATACGAGGATGGGCATGCTCTTCCCG
miR‐22‐3p	**AAGCUGCCAGUUGAAGAACUGU**

*Note*: The bold text represents the sequence of miR22.

#### 2.1.2 Characterization

The successful synthesis of tFNAs and tFNAs‐miR22 was confirmed through rigorous characterization techniques, including Polyacrylamide gel electrophoresis (PAGE) and high‐performance capillary electrophoresis (HPCE) for analysing molecular weight differences, dynamic light scattering particle size analyser (DLS) for determining particle size, zeta potential detection for measuring charges, and transmission electron microscopy for examining morphologies.

### 2.2 Cell models and treatment

RGC‐5 cells were obtained from ATCC and incubated in DMEM/F‐12 medium (1× Dulbecco's Modified Eagle's Medium Nutrient Mixture F‐12 [Gibco, USA]) with 10% fetal bovine serum [FBS (Gibco, USA)] at 37°C, with 95% air and 5% carbon dioxide for 24 h. Then, the cells were cultured under normal (95% air, 5% CO_2_) or Oxygen and glucose deprivation(OGD/R)conditions. The OGD/R conditions involved incubating the cells in a sugar‐free medium with varying concentrations (10, 62.5, 100, 125, 200 and 375 nM) of tFNAs‐miR22, 100 nM miR22, 100 nM tFNAs, 0% oxygen, and 5% CO_2_ for 8 h. After 8 h OGD treatment, change the cell culture medium into DMEM/F‐12 basic supplemented with 10% FBS and placed in a cell incubator at 37°C for 24 h.

### 2.3 Cell viability

#### 2.3.1 Proliferation assay

A CCK8 assay was performed to assess cell proliferation. The optimal dosage of tFNA‐miR22 for the experiment was determined using this assay. Cells in logarithmic growth phase were harvested, counted, and seeded into 96‐well plates at a density of 5 × 10^4^ cells/mL. Each well received 100 μL of cell suspension, with three wells per condition. The plates were then incubated at 37°C with 5% CO_2_ for 16–20 h to allow cell attachment and recovery. Different concentrations of tFNA‐miR22 (10, 62.5, 100, 125, 200 and 375 nM) were added to the respective wells in a final volume of 100 μL per well. Blank medium served as the control well. Subsequently, 10% CCK‐8 was added to each well and incubated for 0.5–4 h. Absorbance at 450 nm was measured using a spectrophotometer after the incubation period.

#### 2.3.2 Cell uptake

To assess cellular uptake of tFNAs and tFNAs‐miR22, we labelled the ssDNA with Cy5 fluorescence. RGC‐5 and HUVEC cells were cultured in a 6‐well plate at a density of 3 × 10^5^ cells per well. After 16 h incubation, Cy5‐tFNAs and Cy5‐tFNAs‐miR22 were added to the cells and incubated for 2, 6, or 12 h. Flow cytometry was then used to measure uptake efficiency by detecting Cy5 fluorescence. This allowed us to analyse the amount of labelled tFNAs and tFNAs‐miR22 taken up by the cells quantitatively.

#### 2.3.3 Cell cycle

After adjusting the cell density to 3 × 10^5^ cells/mL, the cells were seeded into 6‐well plates and incubated overnight at 37°C and 5% CO_2_. The medium was then replaced with sugar‐free medium and tFNAs and tFNAs‐miR22 were added at a concentration of 100 nmol/L. The cells were cultured under anoxic conditions for 8 h before collecting samples for cell cycle analysis using a staining kit (Yeasen) and flow cytometry (Attune NxT instrument, Invitrogen).

#### 2.3.4 Apoptosis

The survival rate of RGCs under different treatments was determined by counting cells using a high‐power light microscope. The control group had a survival rate of 100%, and the data for other treatment groups were expressed as a percentage of the cell count compared to the control group. After cell counting, the density was adjusted to 3 × 10^5^ cells/mL and seeded into 6‐well plates. They were cultured overnight at 37°C with 5%CO_2_. The medium was then replaced with sugar‐free medium and tFNAs, miR22, and tFNAs‐MIR22 were added. The cells underwent OGD/R conditions. Apoptosis samples were collected and stained using AnnexinV‐FITC Apoptosis Detection Kit (Beyotime) as instructed by the manufacturer. Flow cytometry analysed the stained cells using an Attune NxT instrument (Invitrogen).

### 2.4 Immunofluorescence assay

Collect the eyeballs with preserved optic nerves from each experimental group, place them in FAS eye fixative. Remove the anterior segment of the eyeball tissue (cornea, iris, and lens tissues) to prepare an optic cup. Use OCT gel (optimal cutting temperature compound, SAKURA, USA) to embed the optic cup. Then, 5 μm thick slices were prepared using standard frozen microtome (CM1950, LEICA), and sagittal sections (with the optic nerve) of the central part of the retina were taken. After rewarming the frozen sections, 4% paraformaldehyde was used to fix the retinal morphology, followed by washing with PBS for three times. Incubate with Triton X‐100 for 10 min to permeabilize cell membranes. Block slices in BSA blocking buffer for 1 h, then stain with Anti‐Synaptophysin antibody (SYN, Rabbit, 1:1000) and Anti‐RBPMS Polyclonal antibody (RBPMS, Mouse, 1:500). Incubate stained slices overnight at 4°C, wash thoroughly to remove unbound antibodies and other nonspecific bindings. Perform secondary staining using fluorescently labelled secondary antibodies Alexa Fluor 488 AffiniPure Goat Anti‐Mouse IgG (1:1000) and Alexa Fluor 594 AffiniPure Donkey Anti‐Rabbit IgG (1:1000). Ensure that secondary antibodies bind to primary antibodies targeting specific proteins. Fluorescence microscopy was used to visualize the fluorescence signals of SYN and RPBMS.

### 2.5 Western blotting

The retina was separated from the eyeball under a stereomicroscope, and each tube was added with 200 μL of protein extraction buffer (RIPA protein lysis buffer + protease phosphatase inhibitor, 1:50), as well as 2 grinding steel beads with a diameter of 3 mm. The retinal tissue was ground using a high‐throughput tissue grinder until no tissue chunks were visible in the tube. And the supernatant was transferred to a new tube for protein quantification. Using the BCA protein quantification method, the extracted proteins were quantified and balanced, mixed with loading buffer, and loaded onto a 10% SDS‐PAGE gel for electrophoresis. Proteins were separated by size through electrophoresis. The separated proteins were then transferred onto polyvinylidene difluoride (PVDF) membrane and incubated with 5% bovine serum albumin (BSA) solution at room temperature for 1 h to block nonspecific antibodies. Incubated the membrane with the diluted primary antibody at 4°C overnight, Phospho‐p44/42 MAPK (p‐Erk1/2, 1:1000, 4370S, CST), Anti‐Synaptophysin antibody (SYN, Rabbit,1:1000) and Anti‐RBPMS Polyclonal antibody (RBPMS, Mouse, 1:500), Caspase3 (1:500; ab13847, Abcam), Bax (1:1000; 2772S, CST), and Bcl‐2 (1:500; ab196495, Abcam). Add a secondary antibody from the same species as the primary antibody. Detect the marker (Horseradish Peroxidase, HRP) of the secondary antibody using chemiluminescence and visualize the signal of the target protein in a Western Blot imager and capture images. Use GAPDH and β‐Tubulin as internal controls to correct for differences in protein loading. Quantitatively analyse protein signals by comparing the signal intensity of target proteins with internal controls to evaluate their expression levels.

### 2.6 Real‐time fluorescence quantitative PC

The total RNA was extracted and purified with Trizol and RNEasy Plus Mini Kit (BioTeke, WuXi, China). Then, used the cDNA synthesis kit (Takara, Dalian, China) to reverse transcrip‐tion. All target mRNAs were amplified by Quantitative Real‐Time RT‐PCR using SYBR Green I PCR Master Mix. Table 2 lists the corresponding primers, all of which are BLAST search design with GAPDH amplification as the control.

**TABLE 2:** The sequences of PCR primers.

Gene	Primer sequence (5′‐3′)
FTL	Forward: TCCTACACCTACCTCTCTCTGG
	Reverse: TTGAGATGGCTTCTGCACAT
NET1	Forward: GCCTTAGCAGCCTTGATTTGA
	Reverse: CCACGAAGGGTAAACGACTGTA
NUS1	Forward: CGTCTACGACCACCAAGGTAT
	Reverse: CTTCTGGGGACAGCACCTTC
GAPDH	Forward: TGCACCACCAACTGCTTAG
	Reverse: GGATGCAGGGATGATGTTC

### 2.7 Animal models and treatment

Four hundred and forty‐five adult Sprague–Dawley (SD) rats (males weighing 250‐300 g, aged 9–10 weeks) were procured from the animal center of West China Hospital, Sichuan University (Licence number: 20230213001). They were housed in a controlled environment with access to food and water, maintained at a temperature of 22 ± 1°C, humidity of 55 ± 5%, and subjected to a 12‐h light/dark cycle. All experimental procedures in this study received approval from the Animal Care and Use Committee of the Laboratory Animal Research at West China Hospital, Sichuan University.

Animals were randomly and equally allocated to five experimental procedures: retinal flat mount, HE staining, immunohistochemistry, western blot analysis, and vivo EDI‐OCT inspection. Each procedure required 70 rats divided into three groups: control group, RI/R injury group, and intervention group. The intervention group was further subdivided into tFNAs intervention group, tFNAs‐miR22 intervention group, and miR22 intervention group. Four observations were conducted at each time point after injury (2 h, 1 day, 3 days, and 7 days), with five animals used for each observation time (*n* = 5 rats/10 eyes). Self‐contrast was achieved by injecting normal saline into the right eye and the intervention drugs into the left eye. RI/R injury was induced using the previously described procedures.^25^ The rats were anaesthetised with 2% pentobarbital sodium solution. A 33G intravenous infusion needle connected to a saline‐filled instrument was carefully inserted into the eyes' anterior chambers. The intraocular pressure (IOP) was gradually increased from 60 to 110 mmHg, maintained for 60 min, and then slowly decreased back to 60 mmHg.

### 2.8 Retinal haematoxylin–eosin staining and immunofluorescence staining

All the eyeballs were removed, and the optic nerve was cut off with a blade at 1 mm behind the bulbous wall. The ocular cup was routinely fixed, dehydrated, and embedded. The ocular cup specimens were made into three to four successive 3 μm frozen slices through the optic papilla. Parts of the slices were baked, and haematoxylin–eosin staining was per‐formed to observe the changes of retinal histology under a light microscope. The remaining samples were cultured with the diluted primary antibodies at 4°C overnight and treated by histochemical staining, RBPMS (1:500, ab152101, Abcam), SYN (1:1000, ab8049, Abcam), Dapi (1:8, ab104139, Abcam).

### 2.9 Retinal mounts were employed for the quantification of RGCs

The eyeballs were fixed with 4% paraformaldehyde and placed in a PBS solution. The retina was cut off from four quadrants while maintaining its continuity. It was then laid flat on a slide, rinsed, drilled, and sealed. The primary antibody (Brn3b, 1:200, SC‐6026, Santa Cruz) and mouse secondary antibody with green fluorescence (1:1000) were incubated separately. Retinal tiles were observed under a microscope and photos were taken. RGCs counting method: the retina was divided into four quadrants around the optic nipple, and cells showing green fluorescence in each quadrant were counted directly.

### 2.10 EDI‐OCT examination of rats in vivo

In this study, enhanced depth imaging optical coherence tomography (EDI‐OCT) was used to analyse the retina structure in Sprague–Dawley rats. The rat's head was immobilized, and retinal images were captured using an OCT probe after pupil dilation and eye moistening. EDI mode was enabled to optimize imaging of the deep retina and choroid, with device parameters adjusted for better image quality and reduced motion artefacts. We collected multiple B‐scan images of the rat eye, including the macular area. Using OCT software, we quantitatively evaluated retinal thickness in each experimental group and compared changes under different intervention conditions.

### 2.11 Transcriptome RNA sequencing

Clean rat retinal tissue was cryopreserved in RNA protection solution. High‐quality total RNA was extracted from frozen tissue using an extraction kit and its integrity was checked. The mRNA was enriched, fragmented, and the cDNA library was constructed with sequencing adapters added. Library quantification and quality control were performed to meet high‐throughput sequencing platform requirements. After sequencing, data quality control and alignment to the reference genome were done. Transcript assembly, expression analysis, and differential gene screening followed.

### 2.12 Statistical analysis

Statistical analysis was performed using SPSS 25.0 software. The experimental data were derived from repeated experiments and presented as mean ± standard deviation (SD) to ensure reliability and accuracy. One‐way ANOVA was used to explore significant differences between groups under different treatment conditions, with *p* < 0.05 considered statistically significant. For transcriptome sequencing data, we evaluated differential expression significance through deep mining of RNA sequence data and statistical tests. We analysed RNA sequence data from different experimental groups to identify significant differential expression. We also conducted bioinformatics analysis, including functional enrichment analysis, to understand the biological processes and signalling pathways involved. The number of replicates and total samples were clearly indicated for transparency and reproducibility.

## 3 RESULTS AND DISCUSSION

### 3.1 Preparation and characterization of tFNAs‐miR22

**FIGURE 2:**
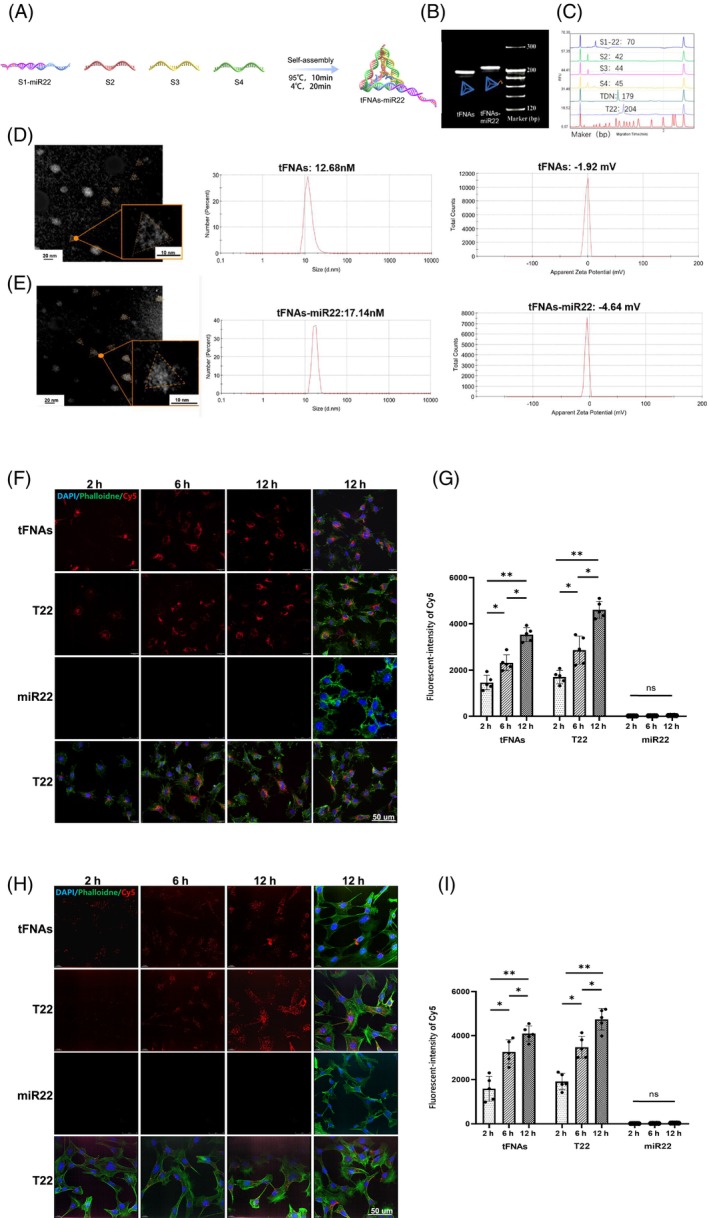
Preparation and characterization of tFNAs‐miR22. (A) Schematic diagram of tFNAs‐miR22 preparation； (B) PAGE shows the successful synthesis of tFNAs and tFNAs‐miR22 and the specific molecular weights. (C) The high‐performance capillary electrophoresis (HPCE) shows the specific molecular weight of S1‐22; S2; S3; S4; tFNAs (TDN) and tFNAs‐miR22 (T22). (D) Left:Transmission electron microscopy (TEM) images of tFNAs (Scale bars = 20 μm); Right:tFNAs were examined by dynamic light scattering (DLS). The microscopic structure of tFNAs is triangular, and the particle size is about 12.68 nm. The ζ potential of tFNAs was ≈ −1.92 mV. (E) Left:Transmission electron microscopy (TEM) images of tFNAs‐miR22 (Scale bars = 20 μm); Right:tFNAs‐miR22 were examined by dynamic light scattering (DLS). The microscopic structure of tFNAs‐miR22 is triangular, and the particle size is about 17.14 nm. The ζ potential of tFNAs was ≈ −4.64 (F–I) Difference in uptake of tFNAs, T22 and miR22 in RGC‐5 and HUVEC (Human Umbilical Vein Endothelial Cells); Red: Cy5‐tFNAs‐miR22; Cy5‐tFNAs and Cy5miR22; Blue: nucleus; Green: cytoskeleton (Scale bars = 50 um); (F) Uptake of tFNAs; tFNAs‐miR22 and miR22 by HUVEC from 2 to 12 h; (G) Statistics of intracellular fluorescence intensity suggest tFNAs accelerated the uptake of miR‐22 in HUVEC; (H) Uptake of tFNAs; tFNAs‐miR22 and miR22 by RGCs from 2 to 12 h; (I) Statistics of intracellular fluorescence intensity suggest tFNAs accelerated the uptake of miR22 in RGC‐5.

tFNAs‐miR22 is formed by combining DNA tetrahedron (tFNA) and miR22 in a 1: (1–4) molar ratio. tFNA consists of four ssDNA strands connected by the preferred DNA sequence (‐TTTTT‐). miR22 is chemically bonded to the 5′ end of S1 (S1‐miR22‐3p, Figure 2A), the sequences of four ssDNA strands and miR22 are selected one‐to‐one (Table 1). The assembly process involves annealing the single strands at 95°C for 10 min followed by cooling to 4°C for 20 min. Successful synthesis of tFNAs‐miR22 was confirmed using 8% polyacrylamide gel electrophoresis (PAGE) analysis. The molecular weight of tFNAs and tFNAs‐miR22 is approximately 200 bp, with the latter being slightly larger (Figure 2B). Then we used high‐performance capillary electrophoresis (HPCE) to verify the successful synthesis of the tFNAs and tFNAs‐miR22. The specific molecular weight of four ssDNA strands (S1‐miR22: 70 bp; S2: 42 bp; S3: 44 bp; S4: 45 bp) and tFNAs and tFNAs‐miR22 is also shown in Figure 2C. Transmission electron microscopy (TEM) images showed the microscopic structure of tFNAs and tFNA‐miR22 is like a triangle, and the results of dynamic light scattering (DLS) indicated that the particle size is 12.68 and 17.14 nm; the zeta‐potential was −1.92 and −4.64 mV (Figure 2D,E).

tFNAs can be combined with small molecule drugs or other bioactive molecules in various ways, such as electrostatic adsorption, sequence extension, and sequence complementarity.^26^ Each of these methods has its own advantages and disadvantages. The method utilized in this study is the sequence extension, which securely links miR22 to the DNA single strand of S1 through chemical bonding. Compared to other drug delivery techniques, this strategy offers the advantage of enhancing the biological stability of miR22 by preventing its degradation from nucleases in the bloodstream while also improving the uptake efficiency.^27^, ^28^ The chemical bonding of miR22 to a single strand of nucleic acid in tFNAs‐miR22 not only optimizes drug release processes, but also allows for control over the timing of miR22 release within lysosomal environments inside cells, ensuring effective drug function.^29^ The stable synthesis of tFNAs‐miR22 was validated by PAGE and HPCE analysis. As expected, tFNAs‐miR22 exhibited a triangular shape under TEM due to its tetrahedral structure. Compared to tFNAs, tFNAs‐miR22 displayed slightly larger particle size and lower ζ potential, indicating consistent structural stability between the two. These experimental results demonstrate the successful construction of stable tFNAs‐miR22 through chemical bonding of miR22 to a single strand of nucleic acid. This provides a reliable foundation for future exploration into treatments and interventions on retinal ischemia/reperfusion injury research.

### 3.2 Uptake of tFNA‐miR22 by RGCs and HUVEC Cells

We compared the uptake rates of three drugs (tFNAs, tFNAs‐miR22, and miR22) in two different cell types (RGC‐5 and HUVEC cells) using confocal microscopy. RGC‐5 and HUVEC cells are important components of retinal neural tissue and vascular tissue, respectively with significant biological differences in their properties and functions. By observing the drug uptake rates in these two cell types, we can assess the biological behaviour of tFNAs and tFNAs‐miR22 in diverse cellular environments. This assessment contributes to determine the uptake efficiency of these drugs in retinal tissue. Figure 2F shows the uptake of these drugs by HUVEC cells from 2 to 12 h, with red fluorescence represents Cy5‐labelled drugs (the first row Cy5‐tFNAs; the second row Cy5‐tFNAs‐miR22; the third row Cy5‐miR22; the fourth row Cy5‐tFNAs‐miR22 with three channels), green fluorescence (Phalloidine) marks the cell skeleton, and blue fluorescence (DAPI) marks the cell nucleus. The results revealed that the uptake of tFNAs and tFNAs‐miR22 significantly increased starting at 2 h and continued to increase from 2 to 12 h. However, the efficiency of miR22 entering cells was lower compared to the other two drugs (Figure 2F,G). Similar trends were observed in RGC‐5 cells (Figure 2H,I).

Fan et al.^30^ used single particle tracking to trace the endocytotic internalization and subsequent transport of tFNAs in cells in detail. They found that tFNAs first enter the cell through a caveolin‐dependent pathway, and then undergo orderly transport within the cell through microtubule‐dependent pathways, finally being successfully delivered to the lysosomes. These tFNAs can maintain structural stability in the cytoplasm for 12 hours.^30^ Previous studies have shown that miR22 can also enter cells through endocytosis or direct transmembrane transport.^31^ However, our results suggest the efficiency of miR22 entering cells is relatively low compared with other two drugs. This may be due to the small size of miRNA molecules and hinders their ability to penetrate cell membranes or possess efficient intracellular uptake capabilities.^32^ In addition, miR22 has poor stability in blood or tissues thereby reducing the effective concentration reaching the target cells.^33^ Unmodified miR22 also lacks sufficient specific binding sites to effectively target cells, resulting in overall low absorption efficiency.^34^ In addition, miR22 lacks stability in blood and tissues thereby reducing its effective concentration reaching target cells.^59^ Unmodified miR22 also does not have enough specific binding sites to effectively target cells, resulting in overall poor absorption efficiency.^60^ Figure 2 demonstrates the excellent cell‐entry performance of tFNAs‐miR22. Whether in RGCs or HUVEC cells, tFNAs facilitate rapid and efficient cellular uptake of miR22. Furthermore, tFNAs provide protection against degradation and exhibit lower immunogenicity compared to other delivery vectors,^19^ thereby significantly enhancing the activity of miR22 in vivo. These findings are essential for establishing tFNAs as a small molecule carrier and for demonstrating the substantial biological impact of tFNAs‐miR22 on RGCs.

### 3.3 tFNAs‐miR22 inhibited RGCs apoptosis and promoted proliferation

**FIGURE 3:**
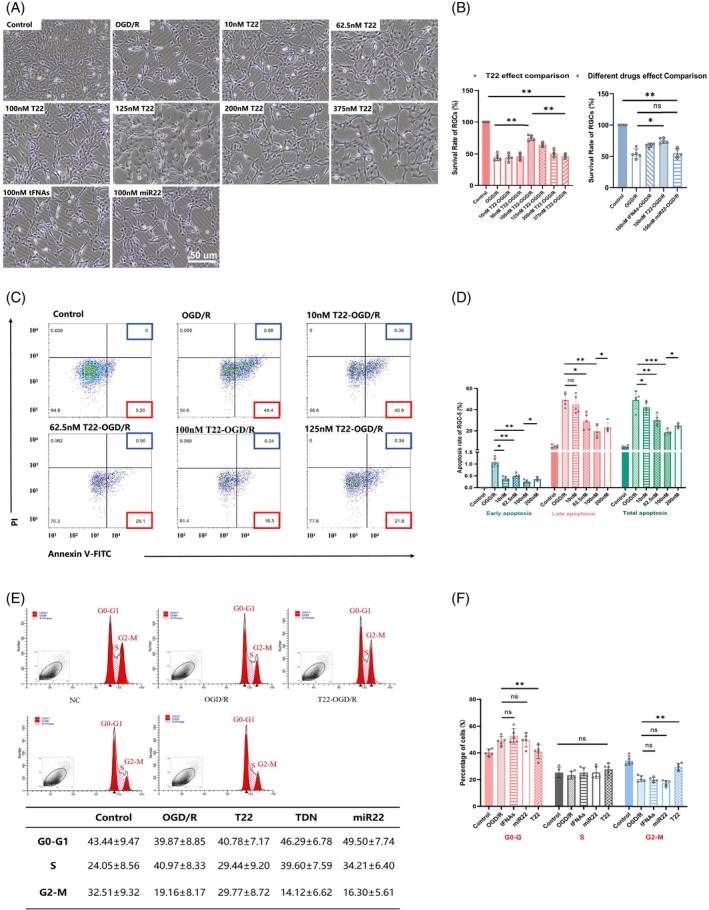
tFNAs‐miR22 increase survival rate and promoted cell proliferation after OGD/R injury of RGCs. (A) The survival rate of RGCs under different treatments was observed under light microscope. Scale bar = 50 μm. (B) A quantitative statistical analysis of survival rate by cell counts under light microscope (The number of cells in the control group was taken as 100%, and the data of each experimental group was expressed as a ratio). (C) The apoptosis rate of RGCs under different concentrations of tFNAs‐miR22 was analysed by flow cytometry. (D) A quantitative statistical analysis of apoptosis rate by flow cytometry. (E) The cell cycle of RGCs under different treatment was analysed by flow cytometry. And a statistical table for the specific data of cell cycle. (F) A quantitative statistical analysis of cell cycle. All data are performed using one‐way ANOVA followed by Tukey correction for multiple comparisons and presented as mean ± standard deviation (SD) (*n* = 5). Statistical analysis: *p < 0.05, **p < 0.01, ***p < 0.001.

Acute RI/R injury can cause rapid and massive loss of RGCs. The ideal therapeutic drugs should penetrate RGCs quickly and effectively reverse their apoptosis. To investigate the optimal concentration of tFNAs‐miR22 for treatment, we established six drug concentration gradients. Subsequently, tFNAs‐miR22, tFNAs alone, and miR‐22 were separately added to normal RGCs and OGD/R injured RGCs, at final concentrations of 10, 62.5, 100, 125, 200 and 375 nM. We added tFNAs‐miR22, tFNAs alone, and miR‐22 to normal and injured RGCs at different concentrations. 100 nM tFNAs‐miR22 effectively reversed RGC apoptosis following IR injury (Figure 3A). Higher (125 nM) or lower (62.5 nM) concentrations of tFNAs‐miR22 also promoted cell proliferation, but not as effectively as 100 nM (Figure 3B). This suggests that 100 nM is the optimal concentration for inhibiting apoptosis by tFNAs‐miR22. Similar trends were observed in the intervention group treated with tFNAs, indicating a significant therapeutic effect under no‐load tFNAs. However, there was no significant treatment trend observed in the miR22 intervention group (Figure 3B Right). Flow cytometry analysis showed that treatment with 100 nM tFNAs‐miR22 and tFNAs had the highest efficacy in promoting RGCs proliferation and inhibiting apoptosis following OGD/R injury (Cell total apoptosis rate was 49.39% in the OGD/R group compared to 18.54% in the intervention group treated with 100 nM tFNAs‐miR22, Figure 3C,D).

To further investigate the impact on the cell cycle, flow cytometry was employed to assess the effects of 100 nM tFNA‐miR22, tFNAs, and miR22 on cell cycle progression in damaged retinal neuron. Results showed that both the control group and tFNAs‐miR22 intervention group had lower cell numbers during the intercellular phase (G0‐G1) compared to other groups. However, expression of miR‐22 was higher in both these groups during G2‐M phase than in other groups (Figure 3E,F). The research results indicate that tFNAs‐miR22 plays a role in cell cycle regulation, leading to most cells being in the G2‐M phase and fewer in the G0‐G1 phase. Previous studies have suggested that tFNAs‐miR22 affects key cell cycle checkpoints by regulating genes related to the cell cycle and may directly promote cells entering the G2‐M phase, thereby increasing cell proliferation.^37^, ^38^ This is consistent with the described effect of tFNAs‐miR22 on RGCs in a simulated glaucoma pathological model experiment,^19^ which showed that tFNAs‐miR22 significantly reduced NMDA‐induced RGCs death. At the same time, tFNAs enhanced the targeted delivery of miR22 to damaged retinal neurons, ensuring precise control of the cell cycle and alleviating OGD/R‐induced damage. The optimal intervention concentration of tFNAs‐miR22 was determined through this experiment, and it was demonstrated that the intervention strategy of tFNAs‐miR22 targeting cell cycle regulation has potential value, providing a new strategy for treating retinal damage caused by RI/R.

### 3.4 tFNAs‐miR22 protects rats retina after RI/R injury

**FIGURE 4:**
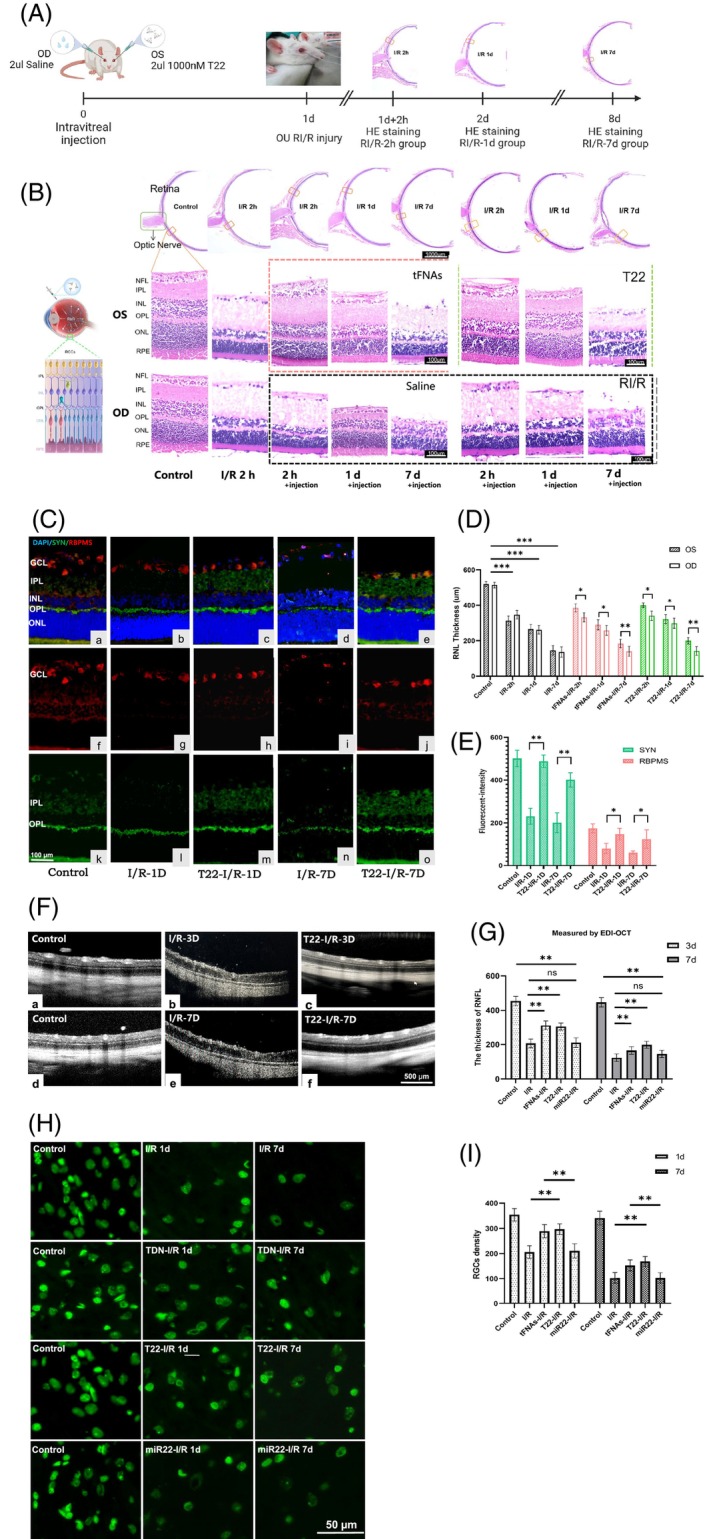
tFNAs‐miR22 protects survival of retinal neurons in rats. (A) An in vivo model of retinal ischemia/reperfusion model and timeline of self‐control experimental operation. (B) HE staining images of rats retinal tissue sections at different magnification. Scale bars = 100 μm. (C) Immunofluorescence staining on cryosections of the retina, RBPMS (active protein of retinal ganglion cells, red fluorescence), SYN (presynaptic vesicle protein, indicated by green fluorescence). Scale bars = 100 μm. (D) Histogram of the retina thickness on HE sections. (E) Histogram of fluorescence intensity. (F) Use Heidelberg EDI‐OCT to observe the retinal thickness and layer in vivo. Scale bars = 500 μm. (G) Statistical analysis of retinal neurosensory layer (RNFL) thickness in vivo. (H) Stretched preparation of retina was used to calculate the density of the RGCs, tFNAs, miR22, and tFNAs‐miR22 treated RGCs at 1 day or 1 week. Scale bars = 50 μm. (I) Statistical analysis of RGCs density. All data are performed using one‐way ANOVA followed by Tukey correction for multiple comparisons and presented as mean ± SD (*n* = 5). Statistical analysis: *p < 0.05, **p < 0.01, ***p < 0.001.

In the preceding section, we validated the neuroprotective effect of tFNAs‐miR22 on OGD/R‐injured RGCs using an in vitro experiment. To further investigate the therapeutic efficacy of tFNAs‐miR22 in vivo, we established the rat RI/R model by creating high intraocular pressure through anterior chamber puncture and administered the drug via intravitreal injection (Figure 4A). Figure 4B OD indicates that synaptic plasticity in IPL occur in the early stage of RI/R injury (within 1 day), leading to an increase in IPL thickness. In the late stage (1 day after RI/R injury), there is a significant decrease in the number of neurons in the retina, accompanied by retinal atrophy.^25^ The normal central retinal thickness in rats is approximately 500 μm, which decreases to 261.94 ± 24.12 μm 1 day after RI/R injury. After 7 days, neuron loss and retinal atrophy stabilize, with a retinal thickness of 136.89 ± 29.64 μm. We used the same method as in vitro experiments to compare the efficacy of various concentration gradients and determined that 1000 nM is the optimal concentration for intravitreal drug delivery. Considering the dilution effect of the vitreous cavity and the presence of various metabolic enzymes and clearance mechanisms in tissues, higher concentrations of intravitreal drugs are needed to improve stability and effectiveness of drugs in vivo.^39^ Experimental results show that intravitreal drug delivery is an effective route of intervention, and tFNAs and tFNAs‐miR22 can stably exist in the vitreous cavity and exert biological effects on the retina. We investigated the impact of tFNAs‐miR22 on different time periods by administering normal saline to the right eye and tFNAs‐miR22 to the left eye. The effect of tFNAs‐miR22 on the retina was assessed at intervals of 2 h, 1d, and 7d post‐I/R injury (Figure 4A). By self‐comparison, in the early stage of RI/R injury, tFNAs‐miR22 and tFNAs alleviated the changes in IPL. In the late stage of RI/R injury, tFNAs‐miR22 and tFNAs effectively delayed the loss of retinal neurons and retinal atrophy. Specifically, under intervention conditions, the retinal thickness was 290.94 ± 28.54 μm and 321.25 ± 25.33 μm at 1 day after injury, while it was 201.52 ± 24.56 μm and 265.65 ± 18.33 μm at 7 days after injury (Figure 4B). Statistical analysis indicated that in the intervened eyes, tFNA‐miR22 and tFNAs resulted in a thicker retina compared to their own control eyes, with less severe levels of atrophy. Furthermore, at both time points of 1 day and 7 days after injury, the intervention effect of tFNAs‐miR22 was superior to using tFNAs (Figure 4D).

Immunofluorescence staining showed tFNAs‐miR22 increased RBPMS expression (active protein of RGCs, red fluorescence) in ganglion cell layer and synaptophysin (SYN) expression (presynaptic vesicle protein, indicated by green fluorescence) in the IPL and OPL after RI/R injury (Figure 4C,E). RBPMS is a protein involved in the survival, synapse formation, and repair of neurons.^40^ The results suggest that tFNAs‐miR22 enhances the biological activity of RGCs for greater survival and functional recovery. The mechanism of action may involve the impact of miR22 on regulating gene expression related to RBPMS.^41^, ^42^, ^43^ SYN, as a presynaptic vesicle protein, plays a crucial role in synapse formation and neurotransmission.^43^ We observed that tFNAs‐miR22 intervention can result in an increase in SYN levels, promoting the formation of new synapses in IPL or OPL and enhancing synaptic activity. This facilitates more frequent signal transmission by neurons and aids in rebuilding connections to restore visual function.^44^, ^45^ The specific mechanism remains unclear.

Using Heidelberg OCT to observe the retinal condition of live rats. As shown in Figure 4F, the normal rat retina appears clear and well‐layered on OCT, with visible cross‐sections of large blood vessels on the surface. The thickness of the retinal nerve epithelial layer was 467.21 ± 27.22 μm (Figure 4Fa). At 3 days after RI/R injury, the retina shrinks and becomes thinner (the thickness of retinal nerve epithelial layer is 198.75 ± 18.78 μm, Figure 4Fb), with disordered layers. At 7 days after injury, progressive shrinkage of the retina occurs (the thickness of retinal nerve epithelial layer is 175.33 ± 20.54 μm, Figure 4Fe), and the diameter of superficial large blood vessels becomes smaller and blurred (Figure 4Fb,e). Both tFNAs and tFNAs‐miR22 show significant effects in repairing retinal layers and alleviating RI/R‐induced retinal shrinkage (at 3 days post‐injury: 305.47 ± 23.52 μm; 302.17 ± 19.67 μm, at 7 days post‐injury: 197.95 ± 17.33 μm; 201.24 ± 19.21 μm), while maintaining the diameter of superficial large blood vessels (Figure 4Fc,f). Statistical analysis provided evidence that tFNAs‐miR22 and tFNAs exhibited statistically significant efficacy in repairing retinal layers and mitigating retinal atrophy following RI/R injury (Figure 4G).

Figure 4H shows the analysis of RGCs cell density in rat stretched retina, calculating the number of RGCs. The normal RGC density in rats is usually between 600 and 800 cells/mm^2^. At 1 day after RI/R injury, the density of RGCs decreased to 406 ± 25 cells/mm^2^. At 7 days after injury, it decreased to 202 ± 17 cells/mm^2^. The tFNAs‐miR22 group reached 597 ± 21 cells/mm^2^ (at 1 day) and 468 ± 19 cells/mm^2^ (at 7 days). The tFNAs group reached 489 ± 26 cells/mm^2^ (at 1 day) and 452 ± 22 cells/mm^2^ (at 1 day). Both tFNA‐miR22 and tFNAs showed significant intervention effects, preventing the death of RGCs after RI/R injury. miR22 did not show a significant intervention effect, with the miR22 intervention group reaching densities of 398 ± 28 cells/mm^2^ and 232 ± 21cells/mm^2^ **(**Figure 4I **)**. Due to the rapid loss of retinal neurons and visual function damage after RI/R injury, current interventions targeting RI/R injury in vivo are not ideal.^46^ Restoring visual function by effectively protecting retinal neurons remains a major research challenge.^15^, ^47^ Our results indicate that tFNAs‐miR22 not only can inhibit the death of retinal nerve cells, but also promote the recovery of the retina. tFNAs‐miR22 opens new avenues for intervention in retinal damage, especially for neurodegenerative retinal diseases, and may develop into a safe and effective intervention drugs or drug carriers.

### 3.5 tFNAs‐miR22 may exerts a protective effect by activating ERK pathway to regulate gene and proteins expression

**FIGURE 5:**
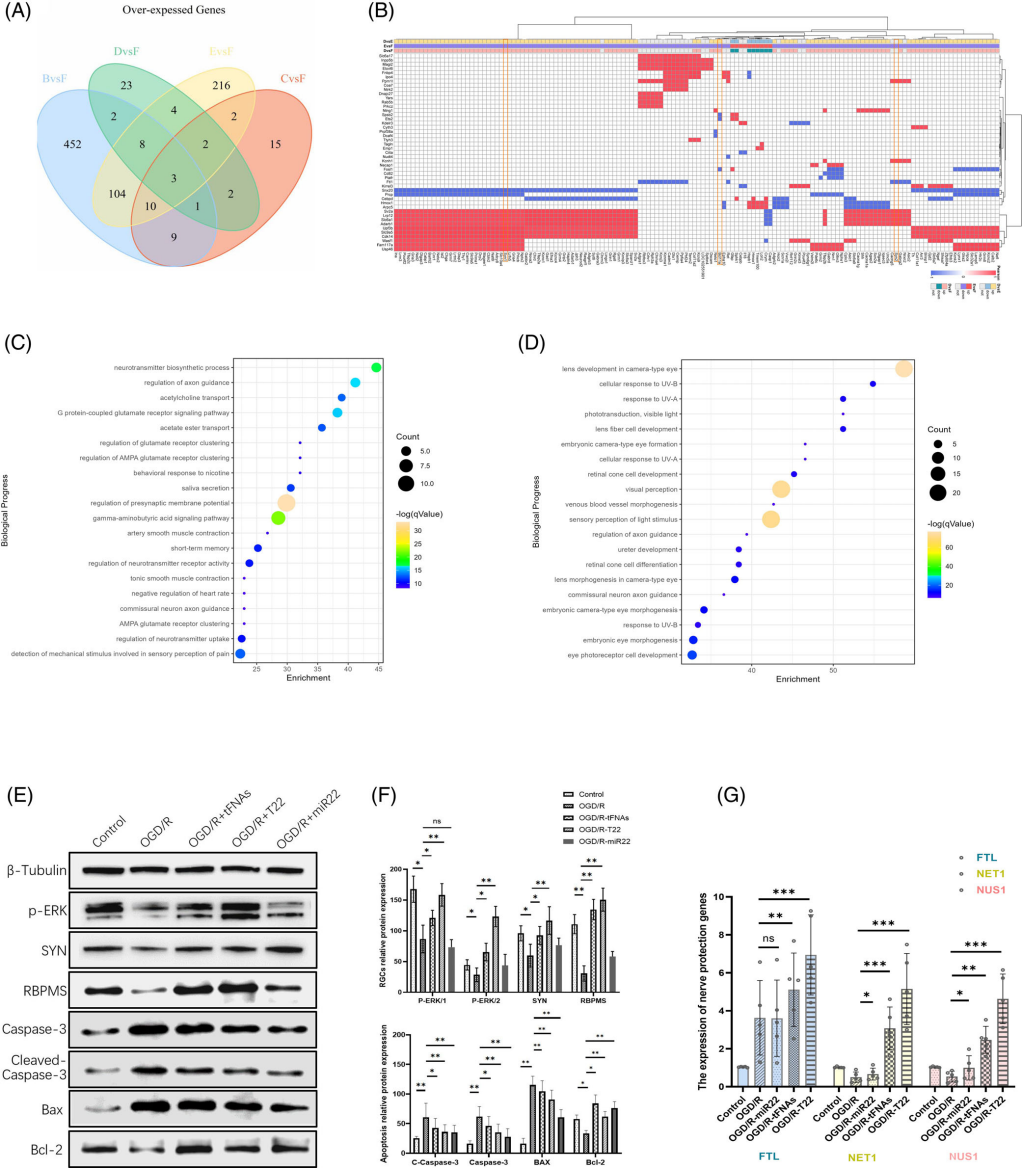
Mechanism of the protective effect of tFNAs‐miR22. (A) The gene expression differences of others were analysed by Venn diagram. (B) The gene expression differences of others were analysed by Heat map [D: tFNAs‐miR22 intervention group; E: Control group; F: RI/R model group]; (C, D) Gene ontology (GO) enrichment analysis showed that the differentially expressed genes between the intervention and model group were significantly enriched in neuro‐related (C) and retina‐related biological processes (D); [X‐axis: targets of miR‐22; Y‐axis: genes that were significantly differentially expressed and enriched in neural‐related pathways between intervention group and RI/R model group]; (E) Western blot was used to detect the expression of proteins related to neuroprotection (F) Statistical plot of proteins expression related to neuroprotection (G) qPCR was used to detect the expression of target factors. FTL, ferritin light chain; NET1, neuroepithelial cell transforming 1 Gene; NUS1, nanoscale ubiquitin‐specific peptidase 1. All data are performed using one‐way ANOVA followed by Tukey correction for multiple comparisons and presented as mean ± SD (*n* = 5). Statistical analysis: *p < 0.05, **p < 0.01, ***p < 0.001.

To further elucidate the protective mechanism of tFNAs‐miR22 on retina, we used RNA sequencing technology to study the differences in gene expression between experimental groups. A comparative analysis of gene expression among different interventions was conducted, and a Venn diagram was generated to show overexpression patterns and interrelationships between these genes (Figure 5A). Based on these results, we selected highly correlated factors for further verification (Figure 5B, Orange square). By Gene ontology (GO) enrichment analysis on the results of transcriptome RNA sequencing, we observed that the differences enrichment of the intervention group was mainly in the neuroprotection‐related, for example, ‘neurotransmitter biosynthetic process’, ‘regulation of axon guidance’, ‘acetylcholine transport’ (Figure 5C), suggesting that tFNAs‐miR22 may affect changes in retinal synaptic transmission after injury. Figure 5D presents the enrichment of differentially expressed retina‐related genes. This contributes to a more comprehensive understanding of the mechanism by which tFNAs‐miR22 impacts the retina, particularly in terms of alterations in gene expression associated with the structure and function of retinal neural tissue.

Based on the bioinformatics analysis of transcriptome RNA sequencing, highly related targets were selected for validation. Combined with previous observations of retinal synapses and neurons after RI/R injury,^48^, ^49^ we further narrowed down the target range to focus on synaptic and apoptosis‐related targets. Western blot analysis showed that under OGD/R injury conditions, the relative expression levels of p‐ERK1/2, SYN, RBPMS, and Bcl‐2 were significantly decreased, while tFNAs and tFNAs‐miR22 led to an expression increase in these proteins. The expression of apoptosis‐related proteins Caspase‐3 and Bax was significantly increased under injury conditions but decreased after tFNAs and tFNAs‐miR22 intervention (Figure 5E,F). In addition, we also detected the expression of target neuroprotection‐related genes FTL, NET1, and NUS1 by qPCR. Figure 5G shows that in the OGD/R group, the expression levels of FTL, NET1, and NUS1 were significantly reduced. However, their expression levels recovered after adding tFNAs and tFNAs‐miR22.

Previous studies have shown that tFNAs‐miR22 can activate the Ras–Raf–MEK–ERK cascade pathway after endocytosis, leading to a significant increase in the phosphorylation level of ERK1/2 protein.^19^ After activation of the Ras–Raf–MEK–ERK pathway, the specific mechanism by which tFNAs‐miR22 exerts its protective effect on retina remains unclear. Our study delves further into this issue and finds that activated ERK1/2 may be involved in the expression of RPBMS in RGCs, upregulating RPBMS protein, thereby helping to maintain the number and activity of RGCs, reduce cell damage, and promote damaged cell repair. Furthermore, P‐ERK1/2 may also play a role in the enhancement of synaptic formation and the upregulation of functional protein SYN. This can lead to an acceleration in the regeneration and remodelling of damaged synapses in retinal neurons, ultimately improving the function of visual signal transmission within the retina. Results show that FTL, NET1, NUS1 factors are upregulated in the retina as neuroprotective genes, the roles of these neuroprotective‐related genes in the retina are summarized in Table 3. Due to the nuclear translocation ability of P‐ERK1/2.^50^ The changes in the above proteins and genes may be related to P‐ERK1/2 nuclear gene regulation,^51^ thereby exerting a protective effect on RI/R‐induced retinal damage.

**TABLE 3:** Description of neuroprotection‐related genes.

Genes	Description	Function in Retina
FTL	Ferritin light chain (One of the main forms of iron storage)	Regulates iron metabolism in the retina to prevent retinal nerve cell damage caused by oxidative stress
NET1	Neuroepithelial Transforming Gene 1	Involves in the development and repair of the retina by regulating the dynamics of the neuronal skeleton
NUS1	Nanoscale Ubiquitin‐Specific Peptidase 1 (A deubiquitinating enzyme)	Involves in the regulation of protein stability related to neuroprotection and synaptic plasticity

The fate of damaged cells depends on the balance between anti‐apoptotic protein (Bcl‐2) and pro‐apoptotic proteins (Caspase‐3, Cleaved‐Caspase‐3, Bax).^52^ P‐ERK1/2 regulates the expression of Bcl family member genes, inhibits the expression of pro‐apoptotic proteins. This modulation effectively maintains the balance between anti‐apoptotic and pro‐apoptotic proteins, ultimately leading to the inhibition of cell apoptosis. In addition, the ERK1/2 signalling pathway also affects downstream targets related to apoptosis (such as Bad, Bim), which can directly affect the binding status of Bcl‐2 and Bax and change their function. Besides the action of P‐ERK1/2, miR22 itself can down‐regulate the expression levels of pro‐apoptotic protein Bax and Caspase‐3, affecting changes in mitochondrial outer membrane permeability and release of cytochrome C,^53^ which are critical steps in the apoptotic process. In conclusion, in the cellular apoptosis network tFNAs‐miR22 may be involved in multiple regulatory mechanisms by influencing the balance regulation of anti‐apoptotic and pro‐apoptotic proteins through different targets, thereby affecting the fate determination of cell death.^54^ The specific regulatory mechanisms await further experimental validation.

Experimental evidence has demonstrated that in conditions of retinal ischemia or hypoxia injury, the expression profile of miRNA within retinal cells may undergo significant changes. This biological adaptive response is aimed at initiating tissue repair and regeneration programs.^55^ Typically, miR22 binds to the 3′‐untranslated region (3′‐UTR) of target mRNA, leading to degradation of the mRNA or inhibition of its translation. Through this negative regulatory mechanism, miR22 can downregulate the expression of specific genes involved in apoptosis and inflammation. This ultimately inhibits excessive cell death and inflammation processes, promoting the functional recovery of damaged neurons.^56^ Although miRNAs are generally considered as negative regulatory factors, some studies suggest that miR22 may promote cellular proliferation, differentiation, migration, and angiogenesis by inhibiting key factors that negatively regulate retinal repair.^57^ In addition, miR22 may regulate key nodal molecules of various signalling pathways, (such as ERK1/2, AKT, NF‐κB) thereby affecting downstream activities in gene networks related to repair and regeneration.^58^ When miR22 is used alone, its small and unstable molecular structure makes it susceptible to degradation or rapid excretion in vivo. This leads to insufficient effective concentration and poor biological effects in damaged retinal cells.^59^ In this study, tFNAs were used as nanomaterial carrier to achieve arriving and internalization of miR22 to retinal cells through surface modification with ligands, increasing local concentration, and enhancing therapeutic effects. By sequence extension, tFNAs can encapsulate miR22 to stable nano‐particle structures, preventing miR22 degradation in the peripheral environment, prolonging its half‐life in vivo, and ensuring its biological activity when reaching retinal cells.^19^ In addition, tFNAs‐miR22 can reduce non‐specific distribution and effects of miR22 on normal tissues, reducing unnecessary systemic toxicity.^60^ The results show tFNAs themselves have a certain therapeutic effect on damaged retina. When combined with miR22, it may produce a synergistic therapeutic effect by fully exerting the regulatory effect of miR22, which downregulates the expression of proteins involved in cell apoptosis (Caspase‐3, Bax), while also upregulating key factors for retinal repair through positive regulation. This enhances the expression of RGCs‐protective proteins (RPBMS and SYN) and neuroprotective genes (FTL, NET1, NUS1), promoting proliferation and activity of retinal neuron. Furthermore, miR22 may also play a role in regulating key molecules within the ERK1/2 signalling pathway,^59^ leading to the activation of the ERK pathway and subsequently impact downstream gene network activities associated with retinal repair and synaptic regeneration, ultimately result in an improvement in overall treatment efficiency.

## 4 CONCLUSION

In summary, we established a simple and efficient delivery system for RI/R treatment by modifying miR22 onto tFNAs. This system has been steadily applied in both in vitro and in vivo RI/R models. Results showed that tFNAs could safely and efficiently deliver miR22 into damaged retinal neurons to impose a neuroprotective effect by inhibiting the loss of damaged neurons and promoting the retinal recovery. More importantly, we also found a synergistic effect between tFNAs and miR22, allowing tFNAs‐miR22 to play a faster and more positive role in the neuroprotection of damaged retinal neurons. tFNAs‐miR22 could activate the ERK1/2 signalling pathway to regulate the expression of RGCs‐protective proteins (RPBMS and SYN) and neuroprotective genes (FTL, NET1, NUS1), and regulate the balance between anti‐apoptotic (Bcl‐2) and pro‐apoptotic (Caspase‐3, BAX) proteins. Therefore, this study not only reveals the potential of tFNAs‐miR22 in retinal injury and repair, but also shows the great advantages of innovative nucleic acid delivery technology in achieving efficient drug delivery, which provides new strategies for the treatment of retinal ischemia/reperfusion injury.

## AUTHOR CONTRIBUTIONS

*Study conception and design*: Yanyan Fu, Xiaoxiao Xu, Yunfeng Lin, Meixia Zhang *Experimental section*: Yanyan Fu, Xiaoxiao Xu, Zhouyuan Yu, Xi Huang, Yinyin Chen, Lina Zhang, Jinnan Liu. *Acquisition of data*: Yanyan Fu, Xiaoxiao Xu, Xiaoxia Xu, Delun Luo, Shiqi Li, Lina Zhang. *Analysis and interpretation of data*: Yanyan Fu, Xiaoxiao Xu, Shiqi Li, Chunyan Lei, Yanyan Fu and Xiaoxiao Xu contributed equally to this work. All authors reviewed the results and approved the final version of the manuscript.

## FUNDING INFORMATION

Meixia Zhang was funded by Chengdu Municipal Science and Technology Bureau Key R&D Support Program, Sichuan Provincial Science and Technology Support (2021ZYD0110) and Project for disciplines of excellence, West China Hospital, Sichuan University (ZYJC21025). Xi Huang was funded by Sichuan Provincial Science and Technology Support (2022YFS0192).

## CONFLICT OF INTEREST STATEMENT

The authors declare no conflict of interest.

## Data Availability

The data that support the findings of this study are available from the corresponding author upon reasonable request.
